# The Role of Liquid Biopsy in the Diagnosis of Oral Squamous Cell Carcinoma: A Systematic Review

**DOI:** 10.3390/ijms27020677

**Published:** 2026-01-09

**Authors:** Piotr Niekra, Paulina Adamska

**Affiliations:** 1 Student Scientific Circle of Oral Surgery, Division of Oral Surgery and Implantology, Medical University of Gdansk, Skłodowskiej-Curie 3C Street, 80-210 Gdansk, Poland; pniekra@gumed.edu.pl; 2 Division of Oral Surgery and Implantology, Medical University of Gdansk, Skłodowskiej-Curie 3C Street, 80-210 Gdansk, Poland

**Keywords:** liquid biopsy, oral cancer, oral squamous cell carcinoma, OSCC

## Abstract

Oral squamous cell carcinoma (OSCC) is one of the most prevalent types of cancer in the oral cavity and head and neck region. Due to its location and psychological and social implications, early detection and treatment are very important. A liquid biopsy can be used to diagnose cancer by analyzing samples of bodily fluids, such as saliva, blood, or urine, for specific molecules released by tumor cells. The objective of this study was to evaluate the use of liquid biopsy in the diagnosis of oral squamous cell carcinoma. A systematic review was carried out, following the Preferred Reporting Items for Systematic Reviews and Meta-Analyses (PRISMA) guidelines (PROSPERO: CRD420251238037). Articles taken into consideration for the review were published before 30 September 2025. The search for manuscripts for the review was conducted using PubMed, Scopus, Google Scholar, and Cochrane databases. Forty-three articles were deemed eligible for inclusion in the systematic review. Key data extracted from the studies included authorship, publication date, study location, methodology, number of participants, and reported complications. Most of the analyzed biomarkers showed promising potential for future use in liquid biopsy for OSCC diagnosis. Tumor DNA and miRNA demonstrated the highest diagnostic accuracy. The standard approach to diagnosis and planning treatment relies on tumor biopsy and diagnostic imaging. Liquid biopsy may complement this process by enabling early detection in high-risk populations and monitoring response to therapy. As such, it serves as a prognostic factor or therapeutic target, successfully identifying disease recurrence.

## 1. Introduction

**Figure 1:**
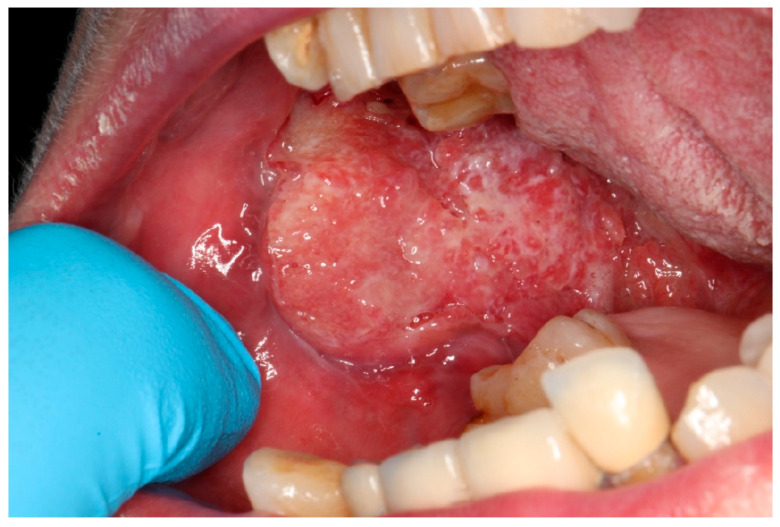
Advanced oral squamous cell carcinoma.

Oral squamous cell carcinoma (OSCC, Figure 1) is a heterogeneous cancer that develops from the mucosa lining of the oral cavity [1]. In 90% of oral cancer cases, patients are histologically diagnosed with OSCC [2], making it one of the most prevalent types of cancer in the oral cavity, accounting for 90% of head and neck squamous cell carcinoma (HNSCC) cases [1]. Data collected by the Global Cancer Observatory (GCO) show 389,846 new cases globally in 2022 for cancer of the lips or oral cavity, with 188,438 patient deaths. It is much more common in males, with incidence (268,999) and mortality (130,808) rates more than double compared to females (120,847 and 57,630, respectively). The age group with the highest number of new cases is 60–74 for females and 55–69 for males [3]. In Poland, the incidence of lip or oral cancer was 2239 in 2022, with 1437 patient deaths. The same tendencies were observed, with more than doubled incidence (1554) and mortality (1019) rates in males compared to females (685 and 418, respectively) [4].

People who exhibit higher alcohol or tobacco use have a greater risk of developing OSCC. The incidence increases fivefold in heavy drinkers, while the promotion of malignant growth in oral cavities is eight times more likely in smokers compared to people with limited or no smoking history [5,6]. Other nicotine products, such as nicotine pouches or toothpowder preparations, also require closer inspection by clinicians due to their increasing popularity. The prolonged use of these products can possibly promote malignant growth on mucosal membranes. HPV types 16 and 18 are also suspected risk factors, but further studies are needed to determine their direct link to the disease [7].

The prognosis of OSCC is mostly poor; patients with aggressive tumors have an overall 5-year survival rate of about 50% [8]. This can decrease to 30% when the patient is diagnosed with advanced clinical stages or at a younger age [9].

The gold standard for oral cancer diagnosis is tissue biopsy with histopathological examination. This method involves either an incisional or excisional biopsy. However, incisional biopsy may not provide a representative sample of tissue; it also has a higher chance of sampling error, thus making the assessment of malignancies harder and easily overlooked [10]. Excisional biopsy removes the entire lesion with a margin of healthy tissue. This makes histopathological analysis easy, while also providing an image of the whole lesion, not just a small part of it.

Liquid biopsy refers to the use of body fluid samples such as saliva, blood (e.g., whole blood, serum, and plasma), or urine to diagnose cancer by identifying specific molecules released by tumor cells. This term was coined in 2010 by Catherine Alix-Panabières and Klaus Pantel, who analyzed the role of circulating tumor cells (CTCs) in blood in detecting breast, prostate, lung, and colon cancers [11]. As the idea developed, a liquid biopsy was used to analyze other bodily fluids and molecules. Due to its non-invasive nature, clinicians have high hopes for its future prospects. It allows for multiple samples to be taken during treatment, while also providing faster results, enabling to monitor the current stage of cancer and act accordingly. Moreover, it has the ability to detect heterogeneity in the tumor. However, it also has its limitations: information is only provided about specific molecules or biomarkers, and a full presentation of the disease is not obtained. Furthermore, the process of isolation and detection is crucial to this method; a lack of technological advancements in this field could possibly hinder its diagnostic capabilities [12]. Another limitation is the lack of laboratory standardization, which makes it harder to compare findings from different laboratories [13].

Biomarkers are molecules found in body fluids that help diagnose the development or recurrence of malignant growth. Some of the most researched examples include circulating tumor cells, circulating tumor DNA (ctDNA), tumor-derived extracellular vesicles (EVs), microRNA (miRNA), long non-coding RNA (lncRNA), and circulating free RNA (cfRNA) [12]. In most cases, their amounts are minimal in body fluids; thus, for the development of this technique, it is important to properly analyze tumor tissue to formulate the most specific diagnostic kit for OSCC. Tumor location also influences the type of body fluid most effective for proper detection. Generally, for OSCC, saliva biopsy is more beneficial, while for other types of HNSCC, blood plasma is a better indicator [14]. Moreover, biomarkers can give either direct information about the existence of malignant growth, as in the case of CTCs, or indirect information, as in the case of miRNAs and their up- or downregulation with oncogenic properties [12,15]. These summarize the challenges faced in developing liquid biopsy into a trustworthy method.

The objective of this study was to evaluate the use of liquid biopsy in oral squamous cell carcinoma.

## 2. Methods

A comprehensive search of the literature was performed following the PRISMA (Preferred Reporting Items for Systematic Reviews and Meta-Analyses) guidelines. The review protocol was registered in the International Prospective Register of Systematic Reviews (PROSPERO) under the identification number CRD420251238037.

### 2.1. Search Criteria

#### 2.1.1. Inclusion Criteria

PICO (P—population, I—Intervention or exposure, C—Comparison, O—Outcome) was used as the search criteria:

P. At least ten patients with oral squamous cell carcinoma;

I. Biomarker testing in body fluids;

C. Standard diagnostic;

O. Biomarker sensitivity and specificity.

The articles selected for the analysis were in English. Only human studies were enrolled in the review process.

#### 2.1.2. Exclusion Criteria

Studies were omitted from the analysis if they failed to fulfill the PICO criteria or did not involve human participants.

### 2.2. Data Collection

A search was conducted of the existing literature in the PubMed, Scopus, Google Scholar, and Cochrane databases. The following MeSH terms were used to search for relevant publications: “liquid biopsy”, “oral cancer”, “oral squamous cell carcinoma”, and “OSCC”. The search was conducted for manuscripts published before 30th September 2025. To scope the available literature, the following inclusion criteria were used: (1) studies using liquid biopsy in the detection of oral squamous cell carcinoma; (2) studies conducted on humans; and (3) studies available in full-text at the moment of conducting the review.

The exclusion criteria were (1) studies that did not utilize liquid biopsy for detection of oral squamous cell carcinoma and (2) pre-prints and commentaries.

### 2.3. Quality Assessment

To ensure a high level of evidence, only studies with at least 10 participants were included in the bias assessment. The risk of bias was evaluated using the Newcastle–Ottawa scale (NOS), which examines three main domains: study selection, comparability of groups, and exposure assessment. The evaluation was conducted independently by the first and second authors. Any discrepancies were resolved through a consensus discussion. The Newcastle–Ottawa scale evaluates selection, comparability, and exposure domains, with a maximum score of 9 stars. Studies scoring ≥7 were considered high quality.

## 3. Results and Discussion

**Figure 2:**
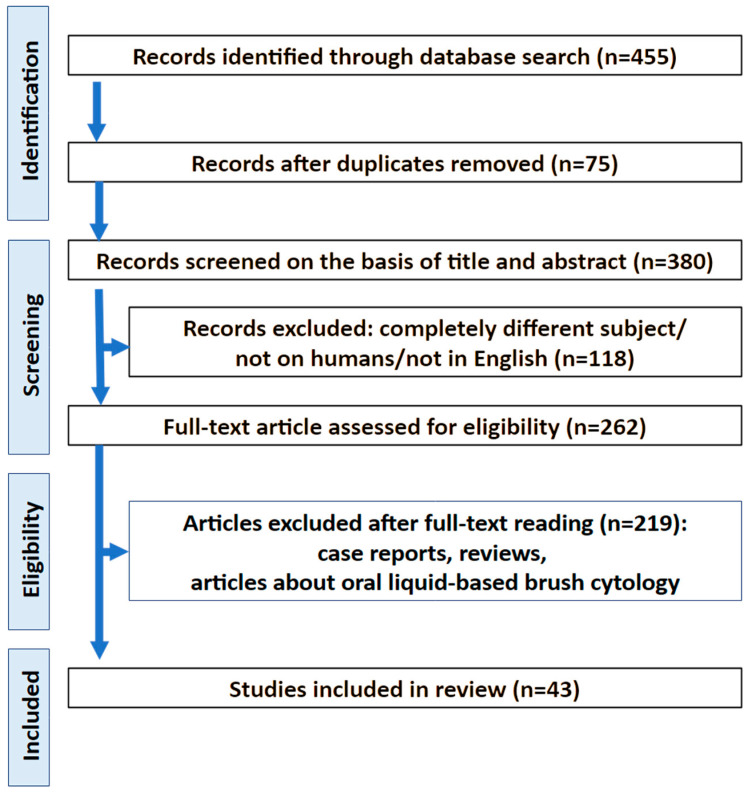
PRISMA flow chart.

The first step of study selection involved screening titles to identify studies that met the predefined inclusion criteria. An initial search across two databases identified 455 records, 380 of which were duplicates. Following abstract review, 43 articles were deemed eligible for inclusion in the systematic review. The selection process is outlined in the PRISMA flow diagram (Figure 2). Key data extracted from the studies—including authorship, publication date, study location, methodology, number of participants, and reported complications—are summarized in Table 1 [14,15,16,17,18,19,20,21,22,23].

### 3.1. Study Characteristics

In total, a liquid biopsy was taken from 2484 patients. Nineteen studies used saliva as the source material, whereas fourteen used plasmas, twenty used sera, and five used whole blood [14,15,16,17,18,19,20,21,22,23,24,25,26,27,28,29,30,31,32,33,34,35,36,37,38,39,40,41,42,43,44,45,46,47,48,49,50,51,52,53,54,55,56]. Studies focused on determining the feasibility of a liquid biopsy as a diagnostic tool for OSCC, with some extending the research to broader fields of HNSCC. The largest study group included 170 patients [47,48].

### 3.2. Risk of Bias

Twenty-five studies were considered to be of good quality, five were moderate quality, and thirteen were low quality. The risk of bias assessment using the NOS is described in Table 2.

### 3.3. Discussion

Our systematic review identified that, in most studies, miRNA is used as a biomarker to detect OSCC. Other diagnostic molecules featured in these studies included lncRNA, cfRNA, shedding of the DNA in mucosal cells, and tumor-specific DNA. The following were properly chosen according to medical knowledge, available databases, or the ability to carry out and compare WES for both healthy and ill individuals. This enabled the creation of OSCC-specific diagnostic kits to assess whether changes in the expression or appearance of certain material can be detected by liquid biopsy, thus allowing for its future use as a diagnostic method.

Tumor DNA is being researched as a possible biomarker. It is constantly released into bodily fluids with shedding cells [57]. Using next-generation sequencing, tumor DNA can be assessed, presenting the option of inventing a cancer-specific panel of mutated genes in the future. According to Ahmed et al. [16], the most frequently mutated genes in the case of OSCC are *TP53*, *FAT1*, *CDKN2A*, *CASP8*, and *DNAH7*. In total, 64% of patients had a mutation in the gene *TP53*, making it the most common marker within the tested group. This is also supported by Wang et al. [14], who identified mutations in *TP53* in 86% of patients. That data was obtained through initial sequencing of tumor samples, which allowed the authors to form a comparison between tumor somatic mutations present in whole DNA (cellular and cell-free DNA) and in saliva. The results showed that 82% of OSCC patients were detected to have identical mutations as sequenced from the tumor sample [30]. Another study, carried out by Shanmugam et al. [46], also featured DNA from saliva, specifically shedding DNA in mucosal cells. In this case, with the help of three independent OSCC WES data sets, mutated genes were also selected [46]. The created panel contained four of the genes from the first study (*TP53*, *FAT1*, *CDKN2A*, and *CASP8*), while also including new genes: *PIK3CA*, *NOTCH1*, and *HRAS*. This panel allowed for the detection of 95.87% of patients with at least one somatic mutation in their saliva. Moreover, it was observed that a smaller panel of only five genes (*TP53*, *CDKN2A*, *FAT1*, *CASP8*, and *NOTCH1*) detected about 93% of OSCC cases in the tested group, underlining the importance of these selected genes, while also implying that the influence of *HRAS* or *PIK3CA* on diagnostic possibilities can be negligible. This requires further confirmation, as a greater number of patients can be detected in a larger test group [46,58,59,60].

It is worth noting that in the case of HNSCC, some DNA mutations occur in the same genes as OSCC, namely *TP53*, *PIK3CA*, *CDKN2A*, and *HRAS* [14]. This might cause difficulties in diagnosing the precise type of cancer when using mutated DNA as a biomarker in liquid biopsy. On the other hand, *PIK3CA* and *HRAS* are suggested to have negligible diagnostic capabilities in terms of OSCC [46], despite being among the most common biomarkers for HNSCC [14]. Moreover, it has been shown that saliva biopsy has a higher detection rate for OSCC [26,43], whereas blood plasma provided better results for other cases of HNSCC. As such, different types of liquid biopsy could be used to detect various types of tumors in the future [14].

MicroRNA is another widely studied biomarker for OSCC detection through liquid biopsy. MicroRNAs are non-coding RNAs that have a significant role in regulating gene expression and are also important in carcinogenesis. They can either induce mRNA degradation through interaction with the 3’ untranslated region of target mRNA or cause activation of translation and regulation of transcription under certain conditions [61]. The expression of miRNA in OSCC varies according to their function; as such, oncogenic molecules are upregulated, while protective molecules are downregulated during the progression of malignant growth. The main miRNAs featured in studies that were significantly downregulated compared to the control were miR-125a [38], miR-139-5p [21], miR-145 [56], miR-3928 [23], miR-138 [43], miR-424 [43], miR-31 [28], miR-200a [38], miR320a [45], miR-758 [20] and miR-106a [52].

MiR-139-5p is suspected of directly inhibiting *HOXA9*. This potential mechanism hinders the proliferation, invasiveness, and migration of OSCC cells [62]. A study by Duz et al. [21] identified that it is a suitable biomarker for detecting a particular type of OSCC: tongue squamous cell carcinoma (TSCC). Authors provided evidence of its sufficient diagnostic accuracy in distinguishing OSCC patients from healthy individuals using an ROC curve with an AUC of 80.5%. Moreover, the research showed that the expression of miR-139-5p returned to normal values 4–6 weeks after the surgical removal of malignant tissue, suggesting the direct suppression of OSCC.

MiR-3928 is another tumor-suppressive molecule that has been assessed as a master regulator of carcinogenesis. For example, in osteosarcoma, its targeting was found to be responsible for cell cycle arrest, bone tissue growth, and immune response [63]. In a study by Farshbaf et al. [23], miR-3928 expression was 67 times lower in OSCC patients compared to healthy individuals. This research also featured a group of patients with oral lichen planus (OLP) for whom the reduction in expression was only six times lower. OLP generally has a low probability of transforming to OSCC, totaling 1–2% of cases [64]. However, the data suggests that the reduction in miR-3928 expression could be correlated with the progression of malignant growth. It is also worth noting that no correlation was found between the grade and stage of miR-3928 expression, indicating that it is an ineffective indicator for further patient monitoring.

Another tumor suppressor is miR-138. By targeting appropriate genes, miR-138 influences cell proliferation, apoptosis, invasion, and migration [65]. It has also been found to sensitize tumors to chemotherapies and, in the case of OSCC, hinder the movement of cells [66]. Rocchetti et al. [43] conducted an investigation into the changing expressions of this miRNA in patients with OSCC and oral potentially malignant disorders (OPMDs). This results showed a significant difference in expression between the three groups (OSCC, OPMD, and control), with slightly decreased expression for OPMD and more significant expression levels for OSCC. Lower levels of miR-138 in both groups may suggest its involvement in early development of malignant tissue, thus indicating its potential as a good early diagnostic biomarker. The authors suggest that miR-138 may be able to regulate the biological processes of OSCC via repressing ISG15 expression.

miR-424 was found to have both cancerogenic and protective functions depending on the tissue [67]. Moreover, its regulation of expression also varies between cancer types. Downregulation was noted, among other observations, for osteosarcoma, intrahepatic cholangiocarcinoma, prostate cancer, and endometrial cancer, while upregulation was observed for melanoma, laryngeal, and esophageal squamous cell carcinomas and glioma [68]. In the case of OSCC, Rocchetti et al. [43] noted that miR-424 demonstrated a significant decrease in expression in the saliva of patients with OPMD and OSCC compared to the control. This data is supported by the research of Ghafouri-Fard et al. [68]. However, their results also include an examination of blood and adjacent normal tissue from OSCC patients, which shows upregulation of miR-424. The reason for decreased expression in the saliva has not been fully studied, but it may be connected to the response of oral tissues to increased expression in OSCC. The results presented by Rocchetti et al. [43] also underline the association between a history of smoking and lower expression of miR-424 in the saliva, possibly suggesting increased expression in malignant tissue, which has been associated with poor differentiation, advanced tumor stages, and cervical lymph node involvement. This is just one of multiple possible mechanisms caused by smoking that affect the expression of this miRNA. Further study comparing the blood and adjacent normal tissue samples of OSCC patients with and without a smoking history needs to be carried out.

miR-106a, on the other hand, was found to have a protective function by inducing the downregulation of LIMK1 expression [47]. This significantly inhibits the proliferation and epithelial–mesenchymal transition of OSCC cells. The results presented by Tarrad et al. [52] indicate significantly lower expression for OSCC patients compared to the control. This is supported by data from Shi et al. [47] and the fact that upregulation of miR-106a suppresses OSCC, while its lowered presence allows for malignant development. The diagnostic accuracy of this biomarker is 80.4% for distinguishing between OSCC and healthy individuals, whereas its accuracy is 60% for distinguishing between grades II and III OSCC [52] and thus it has a relatively moderate power for diagnosing new cases of OSCC. This highlights the fact that, generally, miRNA is ineffective as a distinguishing marker for assessing cancer grade. This is similar in the case of miR-3928, where no grade detection based on expression difference is possible [23].

The next miRNA group consists of miR-106b-5p, miR-423-5p, and miR-193b-3p, which were found to be significantly upregulated, suggesting that their oncogenic properties induce further malignant growth [44]. These were all selected in a study conducted by Romani et al. [44] after global profiling of salivary miRNAs. miR-106b-5p has functions responsible for tumor initiation and progression and contributes to resistance against anti-cancer therapies [69].

Resistance to treatment is also associated with miR-423-5p, which contributes to the emergence of temozolomide resistance in glioblastoma and increased autophagy in hepatocellular carcinoma cells [70]. MiR-193b-3p was found to have a dual nature, either suppressing tumor function, as in case of esophageal squamous cell carcinoma, or causing radioresistance and cancer cell invasion, as in the case of nasopharyngeal cancer [71,72]. The high levels of expression of these three miRNAs were confirmed in almost all saliva samples [44]. Accuracy of those biomarkers was also assessed in diagnosing OSCC using ROC curves. The AUC values were 0.813 for miR-106b-5p, 0.851 for miR-423-5p, and 0.748 for miR-193b-3p. These results oscillated at around 80% diagnostic strength, which was similar to other miRNAs such as miR-139-5p and miR-106a [21,52]. Notably, research also identified the combined distinguishing power of all three miRNAs examined by Romani et al. [44]. This was calculated as AUC = 0.98. Thus, identifying sensitive panels of biomarkers for accurate diagnosis of OSCC from saliva does not involve many molecules or need to be complicated. The proper combination of moderately responsive markers can result in precise distinguishing capabilities overall.

miR-31 has dysregulated expression in multiple cancers. Located on chromosome 9p21.3 near the tumor suppressor genes *CDKN2A* and *CDKN2B*, miR-31 may be downregulated through co-deletion or hypermethylation in several malignancies. In contrast, KRAS mutations can upregulate miR-31 expression in cancers, including OSCC. Although increased miR-31 levels have been reported in tissue and liquid biopsies, data on salivary miR-31 expressions in OSCC patients during postoperative follow-up are limited. This study aims to evaluate salivary miR-31 expression before and after surgery as well as its association with clinicopathological features. Kumari et al. [28] suggest that miR-31 could be used as an adjunct, non-invasive biomarker to assess surgical outcomes in the postoperative surveillance of patients with OSCC.

The human miR-196 family comprises miR-196a1, miR-196a2, and miR-196b, which are encoded by genes on chromosomes 17q21, 12q13, and 7p15, respectively. While miR-196a1 and miR-196a2 share identical sequences, miR-196b differs by a single nucleotide outside the seed region. These miRNAs are located within HOX gene clusters and regulate genes involved in differentiation and oncogenesis. miR-196 is frequently upregulated in various cancers and exhibits oncogenic properties, with increased expression also observed in OSCC cell lines. In the study by Liu et al. [32], increased miR-196a expression was observed in tumor samples, and the TT polymorphism of miR-196a2 correlated with unfavorable survival in patients with OSCC.

A study on miRNA was conducted by Mehdipour et al. [15] featuring miR-146a and miR-155. miR-146a was found to have different functions depending on the type of cancer. It functioned either as oncomir or as a suppressor of tumor progression. It plays a significant role in multiple immune processes, while also taking part in cancer cell metastasis, proliferation, invasion, apoptosis, migration, and cell survival. Data presented by both Mehdipour et al. [15] and Kookli et al. [73] showed no significant change in expression of this biomarker between OSCC and healthy patients. It is worth noting that, in a second study, miR-146a in OSCC induced apoptosis, suggesting its potential tumor-suppressive function in this type of cancer [73]. On the other hand, Mehdipour et al. [15] found significantly higher expressions of this marker in dysplastic OLP and non-dysplastic OLP compared to a control group. This may underline the unexplored nature of miR-146a and its expression, as well as its possible use in the future treatment of this kind of malignancy. However, it is not likely to become a reliable biomarker for diagnosis.

The overexpression of miR-155 is linked to cancerogenic effects in a variety of solid tumors [74]. It was identified that this RNA promotes cancer cell metastasis by causing invasion, migration, and epithelial–mesenchymal transition. Mehdipour et al. [15] found no significant difference in expression levels between saliva of OSCC and control groups. On the other hand, when comparing tumorous tissue of OSCC with paired adjacent cancer-free mucosal tissues, a significant upregulation was noted in the malignant tissue [74]. This emphasizes the need for further investigations on this biomarker, as the only study to have considered miR-155 in liquid biopsy for OSCC detection is the one performed by Mehdipour et al. [15]. This was conducted on a small group of patients (*n* = 15 for each group). Current data on this microRNA does not support its future use as a diagnostic marker.

Long non-coding RNA also has potential as a biomarker for diagnosing OSCC. These RNAs influence the biological behavior of cells and the immune response and transform phenotypes into cells [75]. Furthermore, they have received greater attention from scientists due to their tissue-specific expression patterns [76]. They are widely distributed in body fluids such as blood, urine, and saliva, which is another advantage for their use in liquid biopsy [77]. In the featured studies, only LINC00657 was tested [52]. Also known as NORAD (non-coding RNA activated by DNA damage), LINC00657 has a significant effect by preserving genome stability and cell cycle progression [78]. As a result, its deregulation can cause serious problems and promote malignant growth. Results have shown that seven times more LINC00657 is expressed in OSCC cases compared to controls. Thus, the oncogenic effect of this lncRNA, due to its significant change in expression, enables its use as a diagnostic tool. Another important fact underlined in this study is the high diagnostic accuracy of LINC00657, which correctly distinguishes between grade II and III OSCC in 83.3% of cases. This could not be achieved with miRNA. This suggests that LINC00657 could be used not only as a biomarker for detecting OSCC due to its diagnostic accuracy but also as a marker of a patient’s current oral cancer grade.

Cell-free RNA, also known as extracellular RNA (exRNA), is another type of RNA researched for use in liquid biopsy. It consists of different RNAs, including mRNA, miRNA, lncRNA, and circular RNA (circRNA). cfRNA forms due to cellular activities such as apoptosis, necrosis, and active secretion from the nucleus or cytoplasm [79]. In this way, cfRNA is transferred to local bodily fluids, making it a suitable marker for liquid biopsy. The use of cfRNA for OSCC diagnosis is still in its infancy, and limited research is available on this topic. Hu et al. [26] decided to develop this concept by comparing the expression patterns for OSCC and healthy patients. CLEC2B (C-Type Lectin Domain Family 2 Member B) was markedly upregulated in the OSCC group, whereas F9 (Coagulation Factor IX), DAZL (Deleted in Azoospermia 1), and AC008735.2 were significantly downregulated compared to the control group. CLEC2B is responsible for encoding proteins that are responsible for cell adhesion, cell–cell signaling, and which play a role in inflammation and immune response. lncRNA AC008735.2 is also correlated with immune response and could be used to predict immune checkpoint blockade and patient prognosis using head and neck data from The Cancer Genome Atlas (TCGA) [80]. This correlates with data obtained from gene set enrichment analysis (GSEA), where signals of multiple immune-related pathways increased in saliva of the OSCC group compared to the control. These included neutrophil activation, antigen processing and presentation, myeloid leukocyte activation, and natural killer cell-mediated cytotoxicity. This suggests that immune disorders in the oral environment are caused by OSCC, which also supports the validity of discovered biomarkers.

Liquid biopsy is a method for malignant growth diagnosis, not only for OSCC, but for HNSCC, breast, prostate, lung, and colon cancers. Wang et al. [14] identified that saliva has more effective diagnostic properties for detecting cancer in the oral cavity, as 100% of patients with OSCC were detected with tumor DNA in saliva samples. In other sites, such as the oropharynx, larynx, and hypopharynx, the results were not satisfactory; only 47%, 70%, and 67% of patients were detected, respectively, with tumor DNA in saliva samples. In this case, blood plasma samples were more effective as the detected percentages of patients with tumor DNA were 91% for oropharyngeal cancers, 86% for laryngeal cancers and 100% for hypopharyngeal cancers. This places emphasis on the importance of pairing types of liquid biopsy with the suspected cancer or implementing a comprehensive diagnosis by taking both saliva and blood samples. From the results, 96% of patients who had both samples taken had a tumor-specific alteration detected in at least one biopsy.

The diagnostic potential of liquid biopsy is being investigated not only for the preoperative detection of malignant lesions but also for the early identification of disease recurrence. These findings highlight a promising future direction for the development of liquid biopsy–based tools aimed at the early diagnosis of postoperative relapse. This review is among the first to comprehensively evaluate liquid biopsy as a potential diagnostic adjunct for OSCC by assessing the performance of various investigated biomarkers.

At present, the gold standard for OSCC diagnosis and treatment planning remains tissue biopsy obtained directly from the tumor, which, in combination with diagnostic imaging, allows for a comprehensive clinical assessment. Within this framework, liquid biopsy should be regarded as a complementary rather than a replacement tool. Its greatest potential lies in early detection and monitoring, particularly in high-risk populations, including tobacco and e-cigarette users; individuals infected with HPV; heavy alcohol consumers; users of snuff, betel quid, or pan masala; individuals occupationally exposed to carcinogens; patients with potentially malignant oral disorders (such as leukoplakia, erythroplakia, oral submucous fibrosis, and erosive oral lichen planus); and patients with a history of head and neck cancer. In addition, liquid biopsy may support treatment monitoring, prognostic assessment, identification of therapeutic targets, and detection of disease recurrence.

Despite these promising applications, several limitations must be acknowledged. Many of the included studies were based on relatively small sample sizes and exhibited substantial heterogeneity in biomarker selection, sampling media, and detection methodologies. Furthermore, numerous biomarkers have not been consistently validated across independent cohorts, and many studies relied on single-center designs without external validation, limiting the reproducibility of reported diagnostic accuracies. Standardized cut-off values were rarely provided, and potential confounding factors—such as tumor stage, comorbid oral conditions, smoking status, and inflammatory diseases—were often insufficiently addressed.

In this context, it is important to note that although large-scale diagnostic studies in OSCC, including cytological investigations exceeding 1000 cases, do exist, they remain exceptional. A notable example is a large-cohort cytological study published by Kawaharada et al. [81], which demonstrated the feasibility of such analyses under specific clinical and organizational conditions. However, compared with other organ systems—such as cervical or respiratory cytology, where population-based screening programs and standardized specimen collection are well established—oral cytology and liquid biopsy research face inherent structural and epidemiological constraints. These include lower disease prevalence, the absence of universal screening strategies, heterogeneous clinical indications, and limited access to representative specimens.

Therefore, while large cohorts provide valuable evidence, smaller, well-characterized studies continue to represent a realistic and important source of data in OSCC research. These considerations should be taken into account when interpreting diagnostic performance and sample size limitations in studies evaluating liquid biopsy–based approaches for OSCC.

## 4. Conclusions

Most of the biomarkers analyzed demonstrated promising potential for future application in liquid biopsy and the diagnosis of oral squamous cell carcinoma. Tumor DNA and miRNAs showed the highest diagnostic accuracy. These findings underscore the importance of combining multiple biomarkers to enhance early detection, particularly in high-risk patients. Liquid biopsies can be used to monitor disease, detect recurrence, conduct prognostic assessments, identify therapeutic targets, and evaluate responses to radiotherapy or chemotherapy. However, it cannot replace conventional tissue biopsy or diagnostic imaging when planning treatment.

## Figures and Tables

**Table 1:** General information about studies included in the review.

No	References	Country	No. of Patients (OSCC)	Liquid Biopsy	Conclusions
1	Ahmed et al., 2024 [16]	UK	14	saliva; plasma	The findings contribute further evidence suggesting that salivary DNA assessment may play a significant role in monitoring treatment response and identifying early relapses in OSCC. Detecting recurrence at an early stage could improve the effectiveness of salvage surgical interventions for this cancer.
2	Chang et al., 2018 [17]	Taiwan	112	plasma	Three plasma miRNAs were identified as potential biomarkers for distinguishing OL from OSCC. This miRNA panel indicated high diagnostic accuracy and may be useful for OL monitoring and early OSCC detection, supporting the role of circulating miRNAs in tracking malignant transformation.
3	Chen et al., 2018 [18]	China	121	serum	miR-99a may be a useful biomarker for early detection and prognosis of OSCC.
4	De Sousa et al., 2018 [19]	Brazil	16	whole blood	Proteomic analyses identified LDH to be a key radiotherapy (RT) target under hypoxic conditions. Although hypoxia may counteract the effects of RT in OSCC, RT reduced HIF-1α, miR-210, and LDH levels both in vitro and in vivo, highlighting the need for further investigation of RT effects in blood.
5	Dong et al., 2020 [20]	China	28	serum/saliva	This study showed that miR-758 regulates OSCC progression by modulating COX-2 expression, suggesting its potential role in OSCC pathogenesis and as a therapeutic target.
6	Duz et al., 2016 [21]	Turkey	25	saliva	Saliva is a feasible source for routine TSCC diagnostics, and miR-139-5p may serve as a potential biomarker for early detection of TSCC.
7	Emami et al., 2020 [22]	Iran	50	whole blood	This study shows that miR-155, miR-191, and miR-494 are overexpressed in OSCC, and that Avastin reduces their expression while promoting apoptosis in HN5 cancer cells.
8	Farshbaf et al., 2024 [23]	Iran	31	saliva	Given the possible tumor-suppressive function of miR-3928 in the development of OSCC, this microRNA may serve as a promising biomarker for early diagnosis, screening, and future targeted therapies. Saliva represents a dependable medium for evaluating miR-3928, offering notable practical advantages.
9	Gai et al., 2018 [24]	Italy	21	saliva	Salivary extracellular vesicles (EVs) were isolated using a simple precipitation method. They represent a non-invasive source of miRNAs for OSCC diagnosis, with selected EV-enriched miRNAs showing potential as biomarkers.
10	He et al., 2019 [25]	China	45	saliva	Salivary exosomal miR-24-3p may serve as a diagnostic biomarker for OSCC and supports tumor cell proliferation via PER1 targeting.
11	Hu et al., 2025 [26]	China	10	saliva; plasma	The findings indicate that saliva may be a superior source of liquid biopsy compared to blood (plasma) for detecting cfRNA in OSCC. Salivary CLEC2B, DAZL, F9, and AC008735.2 could represent promising diagnostic biomarkers for OSCC and deserve further exploration.
12	Karimi et al., 2020 [27]	Iran	20	serum	Serum miR-21, miR-24, and miR-29a may serve as biomarkers for OSCC detection and potential therapeutic targets.
13	Kumari et al., 2021 [28]	Pakistan	19	saliva	These data support the use of miR-31 as a non-invasive adjunct marker for postoperative monitoring in OSCC patients.
14	Li et al., 2020 [29]	China	50	serum	Higher miR-223-3p expression and tumor differentiation were associated with improved 3-year survival, indicating that reduced miR-223-3p levels may predict treatment response and prognosis in OSCC patients receiving TPF therapy.
15	Liu et al., 2010 [30]	Taiwan	43	plasma	miR-31 in plasma is a diagnostic biomarker for OSCC.
16	Liu et al., 2012 [31]	Taiwan	45	saliva	Salivary miR-31 shows potential as a biomarker for early diagnosis and postoperative follow-up of oral carcinoma.
17	Liu et al., 2013 [32]	Taiwan	95	plasma	Increased miR-196a expression in tumor samples and the TT polymorphism of miR-196a2 correlate with unfavorable survival in patients with OSCC.
18	Liu et al., 2016 [33]	Taiwan	63	plasma	miR-187* has oncogenic effects in oral cancer and could be used as a diagnostic plasma marker for OSCC.
19	Lu et al., 2014 [34]	Taiwan	90	plasma	Joint evaluation of circulating miR-196a and miR-196b levels may provide a plasma biomarker panel for the early identification of OSCC.
20	Maclellan et al., 2012 [35]	Canada	30	serum	Five miRNAs (miR-16, let-7b, miR-338-3p, miR-223, and miR-29a) showed high diagnostic accuracy, indicating their potential as non-invasive biomarkers for oral cancer detection, particularly when combined with other screening methods.
21	Mahmood et al., 2019 [36]	Pakistan	100	plasma	Plasma miR-21 levels in OSCC patients indicate its potential as a diagnostic and prognostic biomarker.
22	Mehdipour et al., 2023 [15]	Iran	15	saliva	Considering the altered expression of microRNA-146a and microRNA-155 in dysplastic OLP and OSCC, their altered expression may serve as a sign of malignancy.
23	Momen-Heravi et al., 2014 [37]	USA	17	saliva	miR-27b may serve as a useful biomarker for differentiating OSCC patients from other groups, supporting the diagnostic value of salivary miRNA profiles in OSCC.
24	Park et al., 2009 [38]	Serbia	50	saliva	Stable miRNAs are present in both whole and supernatant saliva of healthy individuals, supporting the use of salivary miRNAs for OSCC detection.
25	Pedersen et al., 2018 [39]	Denmark	55	plasma	Plasma miR-30a-5p and miR-769-5p can serve as minimally invasive biomarkers for OSCC diagnosis and monitoring of T-site recurrence.
26	Ries et al., 2014 [40]	Germany	57	whole blood	In OSCC patients, miR-494 and miR-3651 were upregulated, while miR-186 was downregulated. miR-3651 overexpression correlated with lymph node status, tumor grade, and clinical stage, suggesting that altered blood miRNA profiles may enable minimally invasive OSCC screening.
27	Ries et al., 2017 [41]	Germany	54	whole blood	Changes in miR-494, miR-3651, and miR-186 expression were significantly associated with OSCC recurrence, indicating their potential use in a minimally invasive blood test for detecting recurrence.
28	Ries et al., 2019 [42]	Germany	53	whole blood	Reduced miR-3651 expression in OSCC tissue may serve as a diagnostic marker, while its inverse expression in the blood suggests that it has distinct tissue and circulating roles.
29	Rocchetti et al., 2024 [43]	Italy	14	saliva; plasma	Overall, our results showed that liquid biopsy from saliva may be a useful tool for identifying diagnostic molecular biomarkers in OSCC and OPMDs.
30	Romani et al., 2021 [44]	Italy	89	saliva	Salivary miR-106b-5p, miR-423-5p, and miR-193b-3p appear to reliably detect OSCC and differentiate patients based on their risk of recurrence. These markers may hold particular value for screening and monitoring high-risk groups, as well as for providing prognostic insights before surgery.
31	Shahidi et al., 2017 [45]	Iran	15	saliva	Salivary microRNA-320a and hs-CRP may represent convenient non-invasive predictors of dysplastic OLP, while IL-6 can serve as a diagnostic and therapeutic target in both non-dysplastic and dysplastic OLP.
32	Shanmugam et al., 2021 [46]	India	121	saliva	Results demonstrate that liquid biopsy can be used to detect low-frequency tumor-associated mutations in salivary oral rinse specimens collected from patients with OSCC.
33	Shi et al., 2019 [47]	China	170	serum	miR-626 and miR-5100 were independently associated with the prognosis of OSCC, highlighting their potential as prognostic biomarkers.
34	Singh et al., 2018 [48]	India	170	serum	miR-21 is significantly upregulated in OSCC compared to OSMF. Higher miR-21 expression correlates with advanced clinical stages of OSCC and with a history of chewing pan-masala. miR-21 may serve as a potential diagnostic and prognostic biomarker.
35	Sun et al., 2016 [49]	China	104	serum	Serum miR-9 levels were reduced in OSCC and OL patients; lower expression was associated with a poor OSCC prognosis, suggesting its tumor-suppressive role and potential as a biomarker.
36	Sun et al., 2018 [50]	China	80	plasma	Plasma miR-200b-3p may serve as a diagnostic biomarker for OSCC.
37	Tachibana et al., 2016 [51]	Japan	31	plasma	miR-223 acts as a tumor suppressor and circulating miR-223 may serve as a diagnostic biomarker and potential therapeutic target in OSCC.
38	Tarrad et al., 2023 [52]	Egypt	12	saliva	Salivary LINC00657 and miR-106a appear to be promising biomarkers for the diagnosis of OSCC. Salivary LINC00657 demonstrates substantial accuracy in distinguishing OSCC from OPMD, and reduced levels of salivary miR-106a may further indicate the presence of malignancy.
39	Wang et al., 2015 [14]	USA	46	saliva; plasma	Tumor DNA in saliva and plasma is a potentially valuable biomarker for detection.
40	Wen et al., 2020 [53]	China	114	serum	MiR-92b is upregulated in patients with advanced OSCC, which can be used as a marker for induction of chemotherapy and prognostic evaluation of advanced OSCC.
41	Xu et al., 2016 [54]	China	114	serum	Elevated serum miR-483-5p was associated with poorer survival and independently predicted prognosis in OSCC, indicating its potential value as both a diagnostic and prognostic biomarker.
42	Yang et al., 2011 [55]	Taiwan	39	plasma	miR-181 may promote lymph node metastasis by influencing cell migration and could serve as a potential biomarker in OSCC.
43	Zahran et al., 2015 [56]	Saudi Arabia	20	saliva	Assessment of salivary miRNAs, particularly miR-184, may provide a rapid, non-invasive, adjunct tool for detecting malignant transformation in oral mucosal lesions.

DNA—deoxyribonucleic acid; EVs—extracellular vesicles; OL—oral leukoplakia; OLP—oral lichen planus; OPMDs—oral potentially malignant disorders; OSMF—oral submucous fibrosis; OSCC—oral squamous cell carcinoma; TPF—T (Docetaxel/Taxotere), P (Cisplatin) and F (5-Fluorouracil); TSCC—tongue squamous cell carcinoma.

**Table 2:** Risk of bias using the Newcastle–Ottawa scale for quality assessment.

No	References	Sample Selection				Comparability		Exposure/Outcome		Total
		Adequate Case Definition	Representativeness of the Cases	Selection of Control	Definition of Control	Comparability of Cases	Controls Based on the Analysis	Ascertainment of Exposure	Data Completeness	
1	Ahmed et al., 2024 [16]	★	★	★	★	★	★	★	★	8
2	Chang et al., 2018 [17]	★	★	★	★	★	★	★	★	8
3	Chen et al., 2018 [18]	★	★	★	★	★	★	★	★	8
4	De Sousa et al., 2018 [19]	★	-	-	-	-	-	★	-	2
5	Dong et al., 2020 [20]	★	-	-	-	-	-	★	-	2
6	Duz et al., 2016 [21]	★	-	★	★	-	★	★	★	6
7	Emami et al., 2020 [22]	★	-	★	★	-	★	★	★	6
8	Farshbaf et al., 2024 [23]	★	-	-	★	-	★	★	★	5
9	Gai et al., 2018 [24]	★	★	★	★	★	★	★	★	8
10	He et al., 2019 [25]	★	★	★	★	★	★	★	★	8
11	Hu et al., 2025 [26]	★	★	★	★	★	★	★	★	8
12	Karimi et al., 2020 [27]	★	-	★	★	-	★	★	★	6
13	Kumari et al., 2021 [28]	★	-	★	★	-	★	★	★	6
14	Li et al., 2020 [29]	★	-	★	-	★	-	★	-	4
15	Liu et al., 2010 [30]	★	★	★	★	★	★	★	★	8
16	Liu et al., 2012 [31]	★	★	★	★	★	★	★	★	8
17	Liu et al., 2013 [32]	★	★	★	-	★	-	★	-	5
18	Liu et al., 2016 [33]	★	★	★	★	★	★	★	★	8
19	Lu et al., 2014 [34]	★	★	★	★	★	★	★	★	8
20	Maclellan et al., 2012 [35]	★	★	★	★	★	★	★	★	8
21	Mahmood et al., 2019 [36]	★	-	★	★	★	★	★	★	7
22	Mehdipour et al., 2023 [15]	★	-	-	★	-	★	★	-	4
23	Momen-Heravi et al., 2014 [37]	★	★	★	★	★	★	★	★	8
24	Park et al., 2009 [38]	★	★	★	★	★	★	★	★	8
25	Pedersen et al., 2018 [39]	★	★	★	★	★	★	★	★	8
26	Ries et al., 2014 [40]	★	★	-	-	★	-	★	-	4
27	Ries et al., 2017 [41]	★	★	-	-	★	-	★	-	4
28	Ries et al., 2019 [42]	★	★	-	-	★	-	★	-	4
29	Rocchetti et al., 2024 [43]	★	★	★	★	★	★	★	★	8
30	Romani et al., 2021 [44]	★	★	★	★	★	★	★	★	8
31	Shahidi et al., 2017 [45]	★	-	★	★	-	-	★	-	4
32	Shanmugam et al., 2021 [46]	★	★	★	★	★	★	★	★	8
33	Shi et al., 2019 [47]	★	-	-	-	-	-	★	-	2
34	Singh et al., 2018 [48]	★	-	★	★	★	★	★	★	7
35	Sun et al., 2016 [49]	★	★	★	★	★	★	★	★	8
36	Sun et al., 2018 [50]	★	★	★	★	★	★	★	★	8
37	Tachibana et al., 2016 [51]	★	★	★	★	★	★	★	★	8
38	Tarrad et al., 2023 [52]	★	-	-	★	-	★	★	★	5
39	Wang et al., 2015 [14]	★	★	★	★	★	★	★	★	8
40	Wen et al., 2020 [53]	★	-	★	-	★	-	★	-	4
41	Xu et al., 2016 [54]	★	★	★	★	★	-	★	★	7
42	Yang et al., 2011 [55]	★	★	★	★	★	-	★	★	7
43	Zahran et al., 2015 [56]	★	-	-	★	-	-	-	-	2

Star (★) = item present; - = item not present.

## Data Availability

No new data were created or analyzed in this study. Data sharing is not applicable to this article.
